# Inter-rater agreement of a pathophysiology-derived clinical assessment tool for complex regional pain syndrome type I in physiotherapy: an explorative study

**DOI:** 10.1186/s12891-026-10219-y

**Published:** 2026-07-28

**Authors:** Jasmin Erni, Evelyn Baertschi, Juergen Degenfellner, Hannu Luomajoki, Florian Brunner

**Affiliations:** 1 Department of Physiotherapy, Balgrist University Hospital, Forchstrasse 340, Zurich, CH-8008 Switzerland; 2 Department of Health, Institute of Physiotherapy, Zurich University of Applied Sciences, Winterthur, Switzerland; 3 Department of Physical Medicine and Rheumatology, Balgrist University Hospital, Forchstrasse 340, Zurich, 8008 Switzerland

**Keywords:** Complex Regional Pain Syndrome, Physiotherapy, Clinical Assessment Tool, Mechanisms, Clinical Sensory Test Battery, peripheral sensitization, central sensitization, cortical reorganization, vegetative responses

## Abstract

**Background:**

Patients with Complex Regional Pain Syndrome Type I (CRPS) typically exhibit highly variable clinical presentations along with significant and prolonged distress. While the role of physiotherapy is considered important, clear evidence-based treatment guidelines are lacking. For development of such in the future, a subgrouping into their underlying pathophysiological mechanisms has been proposed. The aim of this study was to determine the intertester agreement of physiotherapists using a novel clinical assessment tool for patients with CRPS Type I.

**Methods:**

A total of 20 patients diagnosed with CRPS Type I underwent a clinical sensory test battery, examinations concerning body perception and measurements of vegetative processes. The examination was recorded, rated and evaluated by a total of four raters. The therapists categorized their findings into four distinct categories: peripheral sensitization, central sensitization, cortical reorganization, and vegetative responses. They then estimated the proportions of these categories for each patient. The intertester agreement on the estimations was calculated using the Bhatthacharyya coefficient, involving the assessments of four different therapists.

**Results:**

The agreement coefficient among all raters, determined through a methodology for analyzing compositional data, ranged from 0.75 to 0.93.

**Conclusion:**

The multifactorial pathophysiology and its overlap in CRPS pose significant challenges for physiotherapeutic findings and treatments for these patients. In this exploratory study, we have identified good agreement among our findings and gained valuable insights for clinical practice. Furthermore, we hope that our work will aid clinicians in structuring their management approaches for patients with CRPS Type I.

## Background

Complex Regional Pain Syndrome (CRPS) Type I describes a variety of painful presentations that usually develop on a distal extremity within one month after an initiating noxious event such as trauma, surgery or immobilization [1]. Characteristically the symptoms appear disproportionate relative to the inciting event exceeding the expected clinical course both in magnitude and duration. The clinical presentation of CRPS encompasses a wide range of manifestations, including sensory, vasomotor, sudomotor, motor, and trophic changes [1, 2]. Current research suggests that CRPS represents a multifaceted condition involving several mechanisms, such as an aberrant pro-inflammatory response to tissue injury, disturbances in sympathetically-mediated vasomotor control, and maladaptive changes in both peripheral and central neuronal plasticity [3–5].

As a result of this clinical heterogeneity, the diagnosis is often delayed and solely based on clinical signs and symptoms (Budapest criteria) [6]. Since there is no underlying or causal cure, treatment primarily focuses on alleviating symptoms and encompasses various approaches such as medication, interventions, and rehabilitation methods (such as psychology, physical therapy, and occupational therapy) [1, 7–9].

Despite the consensus regarding the importance of physiotherapy in treating CRPS, there is a lack of detailed strategies for physiotherapeutic interventions documented in the existing literature [10]. An accurate evaluation of CRPS symptoms and their underlying mechanisms is essential for tailoring effective treatment strategies and improving patient outcomes [3, 10]. Although some classification frameworks – such as pain phenotype-based treatment recommendations [11] and conceptual mechanism-informed rehabilitation strategies [12] – have been proposed, the absence of standardized, comprehensive and mechanism-oriented clinical assessment tools specifically designed for CRPS within a physiotherapeutic setting remains a significant gap and hinders the delivery of optimal care.

The aim of this explorative study was to develop a comprehensive clinical assessment tool with a structured framework for evaluating patients with CRPS in a physiotherapeutic setting and to evaluate its agreement. We assigned a percentage weighting into four different pathophysiological mechanisms: peripheral sensitization, central sensitization, cortical reorganization and vegetative response. The findings of this study provide a crucial foundation for future mechanism-oriented physiotherapy approaches, aiding in the advancement of effective treatment strategies for CRPS patients.

## Methods

### Development of a novel clinical assessment protocol

In the existing scientific literature, we have identified four potential pathophysiological mechanisms that are believed to contribute to the development of CRPS: neurogenic inflammation, sensitization, maladaptive neuroplasticity, and dysfunction of the autonomic nervous system. These mechanisms have been extensively discussed in the literature [3–5, 12–18]. Considering these findings alongside our empirical experience gained at Balgrist University Hospital in Zürich, Switzerland, we selected four specific driving mechanisms – peripheral sensitization, central sensitization, cortical reorganization, and vegetative responses –these are consistently reported in literature and can be feasibly assessed and addressed within a physiotherapeutic setting. We are aware that other subclassifications have been proposed [19]; however, they partly rely on biologically distinct features that are not accessible to physiotherapeutic assessment. In addition, beyond CRPS I and II, CRPS with Remission of Some Features, and CRPS-NOS, no other formal subtypes are currently recognized [6]. Using the previously proposed mechanisms as a basis, we have developed a comprehensive clinical assessment that incorporates a clinical sensory test battery (CST) [20], evaluation of body perception, and objective identification of vegetative signs to evaluate their occurrence. The assessment can be easily done in every physiotherapeutic practice, with simple daily items like a cotton swab or toothpick.

### Setting and participants

The participants for this study were consecutively recruited from the outpatient and inpatient clinic of the Department of Physical Medicine & Rheumatology of Balgrist University Hospital, Zurich Switzerland between October 2021 and March 2022. FB performed the screening visit and written informed consent was obtained from all participants.

All participants were adults (age ≥ 18) who met the diagnostic criteria for CRPS Type I of the hand or the foot according to the revised Budapest criteria [6]. Exclusion criteria encompassed patients who had CRPS Type II or another co-morbidity that could damage the somatosensory nervous system (e.g. polyneuropathy, stroke or multiple sclerosis). Patients who were not proficient in the German language or those with any mental health condition could impede their participation (e.g. severe cognitive impairment, schizophrenia or psychosis) were also excluded. No formal screening tools for Depression or Post-traumatic Stress Disorder were applied. The investigator provided repeated verbal information to the participants before initiating the testing protocol.

The study was approved by the Ethics Committee of Zurich (BASEC 2021 − 01640).

### Testers

A group of four experienced physiotherapists specializing in musculoskeletal therapy at Balgrist University Hospital performed the measurements. Their clinical experience ranged from seven to twenty-one years, and all had prior experience with CST in patients with CRPS. Two of the therapists were currently treating patients with CRPS in routine practice, while the other two had prior CRPS experience but had since specialized in other fields. Prior to commencing the assessments, the physiotherapists engaged in a comprehensive discussion reiterating the criteria associated with the pathophysiological drivers.

### Design

An explorative cohort study involving 20 patients was conducted by four examiners. The first examiner (JE) performed all the assessments and recorded them on video, while the other three examiners reviewed the video along with additional information, such as secondary diseases, CRPS Severity Score, duration of symptoms, and the original injury that led to CRPS Type I. Once all the relevant information was gathered, the examiners were instructed to assign a total percentage weighting of 100% to the four mechanisms collectively. However, each examiner was required to designate one major driver. Estimations were based on observed clinical signs, rather than symptoms, as they are considered more objective.

The participants remained anonymous, except for the first rater (JE). Each rater performed their estimations independently, without access to the others’ ratings. Data entry was performed by an independent individual.

### Measurements

#### Disease activity

The first rater assessed disease activity using the CRPS Severity Score (CSS) [21], which is based on patient-reported symptoms and clinician-observed signs according to the Budapest criteria [6] (consisting of 16 items rated as 0 for not present or 1 for present). Higher scores indicate a greater severity of CRPS.

#### Assessment of peripheral and/or central sensitization

To assess sensitization, we conducted a CST based on Zhu et al., (2019) [20], which included tests for dynamic mechanical allodynia as well as Responses to Cold Pain Stimulation (CPS), Heat Pain Stimulation (HPS), Mechanical Pain Stimulation (MPS), Pressure Pain Stimulation (PPS) and Wind-up Ratio. These tests were adapted from concepts used in Quantitative Sensory Testing (QST), but involved single standardized stimuli rather than formal threshold determination.

For allodynia, we asked each patient to identify the area where they experienced the most intense pain. Testing began in this area and progressed proximally toward the trunk (not beyond shoulder or hip), until no further discomfort was reported. For the upper extremity, we included dorsal and palmar aspects of the hand, volar and dorsal forearm, and if necessary, the upper arm. The lower extremity testing included dorsal and plantar aspects of the foot, anterior and posterior lower leg, and if necessary the thigh. In each region, several areas were tested using a standard cotton swab. For each area, three to four rapid, gentle swipes were applied over a distance of 1–2 centimeters (cm) within approximately one second. If the patient reported a pain perception ≥ 1 on an 11-point Numeric Rating Scale (NRS 0–10), the test was considered positive. Due to the potential maintenance of neuronal hyperexcitability associated with allodynia further testing in that region was not conducted.

Following allodynia assessment, we evaluated regions adjacent to, but outside the allodynic area. Although tailored to each patient’s individual allodynia pattern, test sites were selected from anatomically comparable regions across participants. In each anatomical section (hand, forearm, upper arm, foot, lower leg, upper leg), up to three distinct sites were tested using various stimuli assessing dysfunctions of the somatosensory nervous system based on the CST [20] and described below. These sites were mirrored on the contralateral, unaffected side to allow for direct side-to-side comparison. As all testing was conducted by a single rater (due to video recording) this further ensured standardization. Throughout the process, patients consistently rated their pain using the NRS. As with allodynia testing, assessment proceeded proximally until equivalent NRS scores were reported bilaterally. Testing did not extend beyond the shoulder or hip. Rather than classifying responses as positive or negative, we evaluated the magnitude of side-to-side differences to infer the relative contribution of peripheral and/or central sensitization.

The response to Cold Pain Stimulation (CPS) was assessed by applying a plastic tube filled with water cooled to -18° to the skin for ten seconds.

To measure the response to Heat Pain Stimulation (HPS), a plastic tube containing hot tap water (approximately 55° C after ten seconds waiting) was applied to the skin for ten seconds using the same water source for all participants.

To measure the response to Pressure Pain Stimulation (PPS), the examiner applied pressure with her thumb to a muscle belly in the affected area, exerting approximately two or three kilograms of force to depress and whiten the underlying tissue. This pressure was maintained for duration of ten seconds.

If the patient could not tolerate the full ten seconds, the stimulus was removed immediately and pain intensity was recorded at that time. If the patient tolerated the full duration, pain rating was recorded at the end of the stimulation.

The response to Mechanical Pain Stimulation (MPS) was tested using a toothpick, to deliver a single pinprick stimulus.

To determine the wind-up ratio, testing was performed once per anatomical section (as described above) but not within the allodynic region. The patient was asked to rate the pain intensity evoked by a single pinprick stimulus, which required a minimum rating of NRS 1/10. Subsequently, the patient rated the average pain intensity of ten repeated pinprick stimuli delivered at a frequency of one per second within an area of one square centimeter (cm^2^). The wind-up ratio was then calculated by dividing the average pain intensity of the ten stimuli by the intensity of the first stimulus.

All tests of the CST were first performed on the unaffected side and then mirrored on the affected side. Test areas were individually selected for each patient, with up to three locations (except wind- up ratio) assessed per anatomical region.

#### Assessment of the presence or absence of cortical reorganization

The assessment of two-point discrimination using an esthesiometer was conducted in an area where light touch sensation was symmetrical in both the right and left extremities. A repetitive fast stimulus was delivered for duration of two to three seconds, gradually reducing the distance between the two points in 5 to 10-millimeter (mm) increments until the subject could only perceive a single point. The distance was then increased to determine the individual threshold. This test was performed in the most painful area but not in the region with allodynia. Two-point discrimination is frequently used in CRPS research to assess cortical reorganization and changes in tactile processing [15, 22].

Furthermore, the voluntary active movement of the affected limb was carefully monitored. Patients were instructed to actively move each joint of the affected hand or foot through their full range of motion. For the hand, this included finger flexion and extension, wrist dorsi- and palmar flexion, radial and ulnar deviation, and pronation and supination of the forearm. For the foot movements included toe flexion and extension, ankle dorsi- and plantarflexion, as well as inversion and eversion. Movements were first performed with visual feedback and then repeated with eyes closed to assess performance without visual control. Passive mobility was assessed in participants who consented to passive movement. The test was considered positive if (a) passive range of motion exceeded active range of motion (AROM), or (b) AROM with visual feedback exceeded AROM without visual feedback. Additionally, the presence of tremors during the movement or visible deterioration in movement quality without visual feedback was regarded as an indicator of a positive test result.

Body perception was assessed using the Bath Body Perception Disturbance Scale (BBPDS), a 7-item scale designed to measure the extent and specific aspects of body perception disturbance [23, 24]. Questions 1–4 and 6b quantify disturbances on a scale from 0 to 10, while questions 5 and 6a are answered with either Yes (1 point) or No (0 points). For question 7, patients were asked to describe the affected limb pictorially with closed eyes. Based on this description, the examiner prepared a drawing, which was then checked for accuracy and ultimately approved by the patient. The drawing was evaluated on a 3-point scale (no distortion = 0, moderate distortion = 1, severe distortion = 2). The sum of all items’ scores reflects the severity of the disorder, with a maximum score of 57.

Patients also completed a run with the Recognise™ App from the NOI Group, which tests the time and correct recognition of the laterality of a hand or foot. The task was completed in a single session without prior training (although some patients had previous experience with the app). The program chosen was always set to “vanilla” mode, comprising 20 images with a maximum time of 10 s per image. Performance interpretation was based on recommendations from the NOI Group, who suggest aiming for 80% accuracy and an average response time of 1.5–2.0 s per image. Slowed or reduced accuracy in the laterality perception of the affected limb is commonly observed in patients with CRPS [17], and it is also considered among the perceptual disorders [5].

#### Assessment of vegetative changes

The final segment of the video recordings involved assessing vegetative signs. Close-up shots of the adjacent extremities were taken to evaluate color differences and abnormalities in hair and nail growth. To identify sudomotor differences, the examiner gently stroked both extremities simultaneously and provided commentary on the observed clinical signs.

The subsequent examinations were conducted without video recording.

Hand or foot circumferences were compared side-by-side and measured using the figure-of-eight method, a validated technique for capturing limb swelling that demonstrated excellent reliability [25]. It is worth noting that the dominant hand of a healthy individual typically exhibits a slightly larger circumference, approximately 7 mm more [26]. Considering a standard error of measurement of 0.42 cm [25], only differences exceeding 1 cm in the dominant hand and differences exceeding 0.5 cm in the non-dominant hand or feet were considered clinically relevant and indicative of edema.

Skin temperature was measured over the most painful part of the hand or foot using an infrared thermometer obtained from a department store (Migros_MClassic_KontaktloserFieberthermometer). The manufacturer reported an accuracy of +/- 0.2 degrees Celsius. A temperature difference of 1° Celsius was deemed clinically relevant or indicative of a positive finding [4]. Temperature assessment was conducted last to allow the patient’s skin temperature to acclimatize to the room temperature.

Table 1 lists all clinical assessments and their conceptual assignment to pathophysiological mechanism, based on literature and clinical experience. Raters used this as a reference to estimate the contribution of each mechanism, integrating the pattern and strength of findings. No fixed scoring system was used; estimations reflected clinical reasoning rather than a standardized algorithm.

**Table 1:** Overview of all assessments conducted and their corresponding pathophyiological mechanism

	Peripheral sensitiziation	Central sensitization	Cortical reoganisation	Vegetative responses
Allodynia		x		
CPS/HPS	x	x		
PPS	x	x		
MPS	x	x		
Wind-up Ratio		x		
2-PD			x	
Active ROM with/without visual control			x	
Body perception			x	
Tremor/dystonia			x	
Lateralization (Recognise)			x	
Color asymmetry				x
Sudomotor changes				x
Hair and nail growth				x
Edema				x
Skin temperature				x

*Abbreviations*: *CPS* Cold pain stimulation, *HPS* Heat pain stimulation, *MPS* Mechanical pain stimulation, *PPS* Pressure pain stimulation, *2-PD* Two-point discrimination

### Statistical analysis - measure of agreement

We employed the methodology developed by Ningning Chen to evaluate the inter-rater agreement for compositional data [27]. Briefly, this approach is based on Bayesian statistics and was originally developed for immunohistochemical (IHC) assays but is adaptable to our context. As measure of overall agreement, we utilize both the Bhatthacharyya coefficient [28], denoted as $\:\rho\:$_B_(BC), and posterior means of shifts, which indicate how a rater differs from the reference rater. The BC quantifies the overlap between two statistical samples or populations: a value of 1 signifies complete overlap, while a value of 0 indicates no agreement at all. The shifts denoted as δ_1_, δ_2_ and δ_3_are understood as follows: for instance, if δ_2_ is greater than zero, the rater has a higher cutpoint value when distinguishing between categories 2 and 3, and vice versa, compared to the reference rater. A shift close to zero indicates that the rater has good agreement with the reference rater on that cutpoint. Our aim was to explore the overall consistency of interpretation across experienced clinicians using the same assessment data; hence we treated all raters equally and calculated agreement coefficients without privileging one perspective as a standard.

To calculate the BC and shifts, we utilized the web application provided by the author [27]. Since Chen’s model is designed to compare one rater to a reference rater, and we lacked a designated reference rater, we determined the BC’s for all four raters and analyzed the outcomes.

### Sample size

Referring to Table 4.6 from Chen [27], our aim was to determine the approximate sample size needed to detect a percent shift in a category of $\:\pm\:$ 7.4% with a power of around 75%. We selected Population 2 from this table, characterized by an agnostic reference mean vector of (0.25, 0.25, 0.25, 0.25) and an intra-rater variability coefficient, K_p_=10, indicating a relatively high degree of variability. Based on these parameters, the suggested sample size was 30 participants without duplications.

All statistical analyses were carried out using R version 3.6.2 [29], while study data were stored and managed using Redcap versions 6.12.1 to 6.14.1 (REDCap, Vanderbilt University, Nashville, TN, United States).

## Results

Due to time constraints, we were only able to recruit 20 participants with CRPS Type I, but enough for an explorative study. They showed a wide range of symptom durations and severity levels. Among them, 17 were female and 3 were male, with a mean (sd) age of 47 [12] years. Half of the participants had a university degree, while the other half had completed vocational training. Symptom durations ranged from 2 to 72 months, with a mean (sd) of 20 [22] months, and CSS ranged from 6 to 17 with a mean (sd) of 11 [3]. Fourteen of the participants were fully work-disabled. All 20 patients underwent the entire testing procedure without any adverse events. Descriptions of the cases and findings from the examinations can be found in Tables 2 and 3.

**Table 2:** Descriptions of the cases

ID	Age	Gender	Work ability	Body part	Affected side	Duration of symptoms (in months)	Occurence
1	60	female	disabled	hand	right*	21	fracture
2	33	female	full	hand	left	6	fracture
3	34	female	disabled	hand	left	18	fracture
4	54	female	disabled	foot	left	3	sprain
5	62	male	disabled	hand	left	3	fracture
6	52	female	disabled	foot	right	23	sprain
7	48	female	full	foot	left	23	sprain
8	46	male	partially	foot	right	15	surgery
9	46	female	disabled	foot	left	72	surgery (2015) sprain (2021)
10	60	female	full	hand	right	2	fracture
11	57	female	disabled	hand	right	9	fracture
12	37	female	full	hand	right*	62	bite
13	45	female	disabled	hand	right*	5	surgery
14	25	female	disabled	foot	right	2	sprain
15	66	female	partially	hand	left	14	fracture
16	33	female	partially	hand	right*	70	fracture
17	43	female	partially	hand	left	22	surgery
18	45	female	disabled	hand	right*	18	fracture
19	50	female	partially	hand	left	10	fracture
20	28	male	disabled	foot	left	3	sprain

Personal information of all participants with affected bodypart, duration of symptoms and occurence of CRPS Type I

*means dominant side affected

**Table 3:** Findings of examination

ID	CSS	Allodynia	CPS aff./(unaff.)	HPSaff./(unaff.)	PPS aff./(unaff.)	MPS aff./(unaff.)	Windup Ratio	Sensory loss	tpd_diff_mm affected side	Arom loss	Motor changes	Edema asymmetries	Trophic changes	Sweating changes	Skin color asymmetries	Temp. asymmetries	Recognise Left/Right accuracy time	Body Awareness changes*	BBPDS Score
1	7	no	2 (1)	2 (0)	0 (0)	2 (1)	3 (1)	no	+ 4	no	no	no	no	no	no	no	80 /90% 2.8 /2.1 s	moderate	15
2	10	no	5 (0)	6 (0)	1 (0)	4 (2)	1.3 (2)	yes	+44	yes	yes	no	no	no	no	no	80 /90% 4.3 / 6.8 s	moderate	22
3	8	no	0 (0)	0 (0)	0 (0)	2 (0)	5 (2)	no	+6	yes	yes	no	no	no	no	no	80 /90% 1.2 /2.9 s	moderate	16
4	14	yes	4 (0)	0 (0)	2 (0)	3 (0)	5 (1)	no	n/a	yes	yes	yes	yes	no	yes	no	100 /100% 1.9 /2.4 s	moderate	21
5	11	yes	0 (0)	0 (0)	0 (0)	0 (0)	4 (2)	no	n/a	yes	no	no	yes	no	yes	no	100 /90% 3.4 /2.4 s	moderate	5
6	12	yes	7 (3)	0 (0)	3 (3)	3 (1)	1 (3)	yes	-25	yes	yes (yes)	yes	no	no	no	no	100 /100% 1.0/ 0.9 s.	severe	25
7	10	yes	10 (0)	3 (0)	8 (0)	10 (4)	2.5 (2.5)	no	+12	no	no	yes	no	yes	no	no	100 /100% 1.6 /1.6 s	severe	28
8	15	yes	2 (0)	3 (0)	2 (0)	3 (1)	2.4 (4)	no	-9	yes	yes	yes	yes	no	yes	no	90 /90% 2.3 /2.2 s	moderate	15
9	17	yes	0 (0)	0 (0)	0 (0)	5 (0)	1.4 (1)	no	+6	yes	yes	yes	yes	yes	yes	yes	70 /70% 2.1/1.8 s	moderate	20
10	13	yes	0 (0)	0 (0)	0 (0)	3 (0)	1.3 (1)	no	+3	yes	yes	no	no	no	yes	yes	80 /100% 1.4 /3.0 s	moderate	12
11	8	no	3 (0)	0 (0)	4 (0)	4 (0)	3 (1)	yes	+5	yes	no	yes	no	no	no	no	60 /70% 3.0 /4.0 s	moderate	19
12	9	yes (yes)	0 (0)	1 (0)	0 (0)	2 (2)	1.5 (2.5)	no	-17	no	no	no	no	yes	no	yes	100 /90% 1.4 /1.1 s	moderate	17
13	12	no	0 (0)	6 (0)	3 (0)	1 (0)	1 (2)	no	+3	yes	yes	no	no	yes	no	no	90 /70% 3.3 /2.2 s	moderate	23
14	11	yes	2 (0)	4 (0)	2 (0)	7 (0)	1.3 (1)	no	+19	yes	yes	yes	no	no	no	no	100 /100% 1.7 /1.9 s	severe	29
15	6	no	0 (0)	0 (0)	0 (0)	2 (0)	2 (1)	no	+2	yes	no	no	no	no	no	no	40 /60% 4.2 /3.6 s	moderate	11
16	11	yes	3 (0)	4 (0)	2 (0)	2 (0)	2 (1)	no	-2	no	no	no	no	no	no	no	80 /60% 4.1 /2.2 s	moderate	20
17	7	no	3 (0)	2 (0)	0 (0)	4 (1)	6 (4)	no	-2	no	no	no	no	no	no	no	80 /80% 4.4 /3.8 s	no changes	13
18	10	yes	2 (0)	3 (0)	7 (0)	4 (0)	5 (1)	no	-42	yes	yes	no	no	no	no	no	70 /100% 2.3 /2.4 s	moderate	10
19	8	no	0 (0)	0 (0)	0 (0)	0 (0)	2.5 (2)	no	n/a	no	yes	no	yes	no	no	no	100 /100% 2.8 /3.1 s	moderate	20
20	15	yes	6 (1)	n/a (0)	2 (0)	4 (0)	2.5 (1)	yes	+11	yes	yes	no	yes	yes	yes	no	80 /80% 2.6 /2.6 s	moderate	21

Findings of all conducted assessments, with scores on the most painful area (not in allodynia or sensory loss areas) at the affected side and non-affected side in brackets or side differences and asymmetries respectively

*Abbreviations*: *aff* affected, *arom* active range of motion, *BBPDS* Bath Body Perception Disturbance Scale, *CSS* CRPS Severity Score, *CPS* Cold Pain Stimulation, *HPS* Heat Pain Stimulation, *MPS* Mechanical Pain Stimulation, *PPS* Pressure Pain Stimulation, *tpd_diff_mm* Two-Point-Discrimination difference in milimeters on affected side, *unaff* unaffected

* ‘moderate’ indicates differences in shape or size compared to the unaffected side, while ‘severe’ indicates the absence of one or more body parts [21]

Table 4 provides an overview of the therapist’s estimations for each participant.

**Table 4:** Weightings

ID	Rater	Peripheral	Central	Cortical	Vegetative
1	A	25%	35%	33%	7%
	B	25%	35%	30%	10%
	C	33%	33%	34%	0%
	D	5%	45%	32%	18%
2	A	20%	30%	40%	10%
	B	10%	50%	40%	0%
	C	60%	30%	10%	0%
	D	7%	31%	44%	18%
3	A	15%	20%	55%	10%
	B	10%	10%	30%	50%
	C	10%	20%	40%	30%
	D	11%	24%	26%	39%
4	A	15%	35%	20%	30%
	B	0%	60%	10%	30%
	C	25%	40%	10%	25%
	D	8%	45%	12%	35%
5	A	0%	30%	25%	45%
	B	5%	40%	25%	30%
	C	0%	40%	50%	10%
	D	18%	35%	5%	42%
6	A	10%	25%	55%	10%
	B	0%	40%	50%	10%
	C	30%	50%	20%	0%
	D	5%	30%	45%	20%
7	A	40%	28%	30%	2%
	B	20%	25%	35%	20%
	C	0%	50%	30%	20%
	D	2%	65%	20%	13%
8	A	20%	20%	20%	40%
	B	50%	10%	10%	30%
	C	10%	40%	20%	30%
	D	16%	28%	11%	45%
9	A	10%	20%	30%	40%
	B	10%	20%	30%	40%
	C	20%	18%	12%	50%
	D	23%	10%	11%	56%
10	A	30%	35%	15%	20%
	B	10%	15%	45%	30%
	C	10%	0%	30%	60%
	D	10%	21%	27%	42%
11	A	35%	30%	25%	10%
	B	10%	10%	45%	35%
	C	35%	12%	45%	8%
	D	18%	15%	39%	28%
12	A	0%	60%	25%	15%
	B	5%	50%	40%	5%
	C	0%	50%	20%	30%
	D	10%	45%	18%	27%
13	A	25%	25%	35%	15%
	B	5%	30%	35%	30%
	C	50%	5%	40%	5%
	D	7%	18%	50%	25%
14	A	25%	20%	50%	5%
	B	10%	30%	55%	5%
	C	10%	60%	30%	0%
	D	13%	23%	48%	16%
15	A	20%	50%	30%	0%
	B	20%	35%	40%	5%
	C	0%	49%	51%	0%
	D	45%	30%	19%	6%
16	A	20%	40%	30%	10%
	B	20%	40%	30%	10%
	C	10%	50%	30%	10%
	D	10%	34%	30%	26%
17	A	45%	40%	10%	5%
	B	30%	50%	20%	0%
	C	0%	51%	49%	0%
	D	31%	37%	16%	16%
18	A	20%	45%	30%	5%
	B	5%	50%	35%	10%
	C	0%	60%	40%	0%
	D	10%	50%	20%	20%
19	A	0%	0%	60%	40%
	B	5%	5%	55%	35%
	C	0%	0%	55%	45%
	D	10%	5%	40%	45%
20	A	0%	40%	35%	25%
	B	5%	30%	35%	30%
	C	5%	40%	35%	20%
	D	5%	40%	25%	30%

Results of the rater’s estimations for each participant, categorized by their respective mechanisms

Table 5 presents estimates for shifts δ1, δ2 and δ3, as well as Bhattacharyya coefficients (BC), alongside credible intervals, contingent on the reference rater. The BCs range from approximately 0.75 to 0.93, denoting a certain degree of similarity or agreement among raters regardless of the reference rater, with values near 1 indicating high agreement. Raters A, B and C demonstrate relatively strong agreement with each other, displaying BC-coefficients exceeding 0.85. Conversely, agreement with Rater D appears to be somewhat lower, with a BC-coefficient of 0.749. Credible intervals are comparatively wide due to the small sample size.

**Table 5:** Results

Reference rater A	Rater	Estimate	Lower	Upper	Reference rater C	Rater	Estimate	Lower	Upper
Shift 1 / δ1	B	-0.558	-1,032	-0.077	Shift 1 / δ1	A	0.183	-0.246	0.533
Shift 2 / δ2	B	-0.315	-0.62	-0.029	Shift 2 / δ2	A	0.054	-0.228	0.36
Shift 3 / δ3	B	-0.237	-0.591	0.198	Shift 3 / δ3	A	0.121	-0.187	0.446
Bhattacharyya		0.858	0.731	0.977	Bhattacharyya		0.934	0.814	0.999
Shift 1 / δ1	C	-0.344	-0.739	0.02	Shift 1 / δ1	B	-0.276	-0.744	0.075
Shift 2 / δ2	C	-0.076	-0.366	0.179	Shift 2 / δ2	B	-0.252	-0.504	0.01
Shift 3 / δ3	C	0.045	-0.423	0.376	Shift 3 / δ3	B	-0.188	-0.504	0.19
Bhattacharyya		0.913	0.808	0.998	Bhattacharyya		0.915	0.815	0.987
Shift 1 / δ1	D	-0.474	-0.918	-0.118	Shift 1 / δ1	D	-0.242	-0.683	0.257
Shift 2 / δ2	D	-0.284	-0.596	-0.027	Shift 2 / δ2	D	-0.24	-0.578	0.089
Shift 3 / δ3	D	-0.661	-0.979	-0.271	Shift 3 / δ3	D	-0.613	-0.917	-0.24
Bhattacharyya		0.749	0.551	0.937	Bhattacharyya		0.781	0.597	0.958
**Reference rater B**					**Reference rater D**				
Shift 1 / δ1	A	0.326	-0.046	0.762	Shift 1 / δ1	A	0.391	0.032	0.796
Shift 2 / δ2	A	0.245	-0.034	0.516	Shift 2 / δ2	A	0.273	-0.062	0.574
Shift 3 / δ3	A	0.34	-0.109	0.74	Shift 3 / δ3	A	0.764	0.367	1,061
Bhattacharyya		0.896	0.776	0.997	Bhattacharyya		0.78	0.607	0.899
Shift 1 / δ1	C	0.033	-0.387	0.499	Shift 1 / δ1	B	-0.061	-0.465	0.388
Shift 2 / δ2	C	0.159	-0.133	0.432	Shift 2 / δ2	B	-0.02	-0.324	0.235
Shift 3 / δ3	C	0.321	-0.129	0.68	Shift 3 / δ3	B	0.486	0.131	0.789
Bhattacharyya		0.921	0.828	0.999	Bhattacharyya		0.856	0.736	0.964
Shift 1 / δ1	D	-0.105	-0.483	0.437	Shift 1 / δ1	C	0.066	-0.467	0.483
Shift 2 / δ2	D	-0.055	-0.341	0.29	Shift 2 / δ2	C	0.188	-0.068	0.486
Shift 3 / δ3	D	-0.405	-0.779	-0.033	Shift 3 / δ3	C	0.735	0.312	1,067
Bhattacharyya		0.853	0.673	0.993	Bhattacharyya		0.804	0.663	0.928

Results of calculated Shifts and Bhattacharyya coefficient, determined for all raters. Shift 1 means the cutpoint between peripheral and central sensitization; Shift 2 the cutpoint between central sensitization and cortical reorganization and Shift 3 the cutpoint between central sensitization and vegetative responses. A shift close to zero means good agreement with the reference rater, while Bhatthacharyya coefficient of 1 signifies complete overlap between two statistical samples

## Discussion

**Fig. 1:**
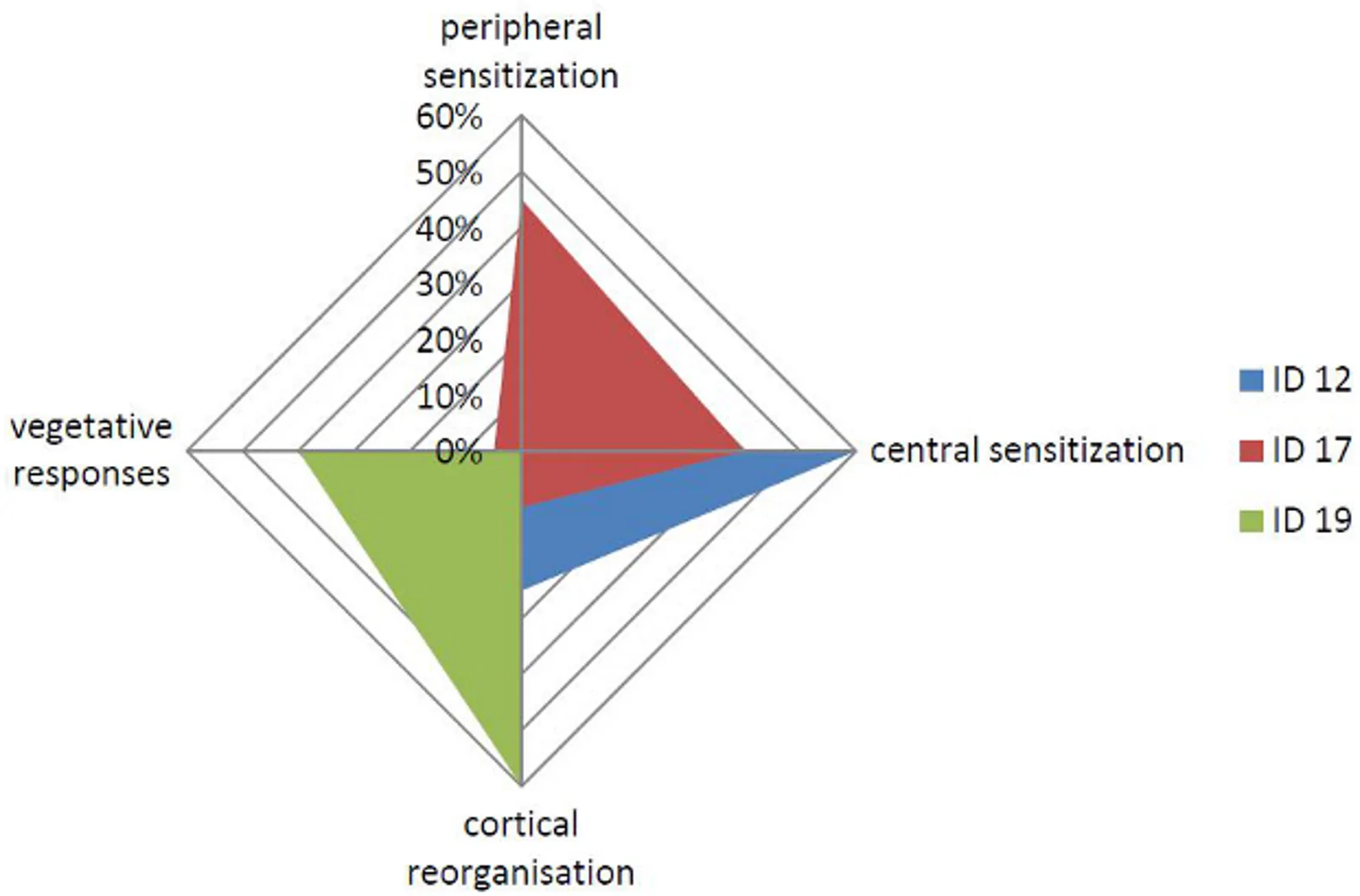
Spider diagram. A spider diagram illustrating how different patients with CRPS Type I can present clinical signs based on the four defined mechanism

The aim of this study was to assess the agreement among physiotherapists regarding a novel clinical assessment tool for patients with CRPS Type I. The results demonstrate good agreement regarding the application of individual weighting across four different mechanisms. There is a growing discussion, that a one-size-fits-all treatment is not appropriate for individual patients in various fields of physiotherapy, and management of pain conditions should be mechanism-based [30–32]. Therefore, subgrouping in patients with conditions such as neck or low back pain, as well as for patellofemoral pain or entrapment conditions, is suggested [33–35]. In our study, we used an estimation to evaluate the extent of the different pathophysiological mechanisms. Although each rater had to determine one dominant mechanism for every participant, the evaluations often did not clearly emphasize any mechanism. However, our pathophysiology-derived clinical assessment may contribute to a personalized physiotherapeutic treatment for patients with CRPS Type I. Inspired by Schmid et al. [36]. we adapted their spider diagram for our clinical research to illustrate how different patients with CRPS Type I can present in physiotherapy (see Fig. 1).

Despite the good results in agreement coefficients, our study pointed out several difficulties encountered when attempting to weigh the pathophysiological drivers in CRPS Type I. Given the complex nature of CRPS, determining the significance of each mechanism proved challenging, particularly in the absence of an established guideline. Considering the difficulties discussed below, the next step should involve reaching a consensus on which assessments are most relevant for each driver and how strongly positive findings should be weighted.

### CST / peripheral sensitization

In retrospect, the definition of allodynia was not entirely clear at the beginning. Allodynia is theoretically described as occurring when a normally non-painful stimulus triggers pain in the person tested [37]. However, this determination proved challenging in practice. Several participants described sensations such as ‘weird’, ‘more sensitive’, or ‘uncomfortable’, making it difficult to classify their experience definitively as allodynia. Additionally, since the participants’ faces were not visible to raters B, C, and D in the videos, they were unable to observe facial expressions, unlike rater A, potentially influencing their judgments. Moreover, participants often reported perceiving stimuli much more strongly on the affected side than on the non-affected side, without finding them unpleasant. This raised questions about whether this phenomenon was hyperalgesia, hyperesthesia related to peripheral sensitization, or indicative of central problems such as a lack of inhibition of descending pathways or cortical reorganization. Hyperalgesia, by definition, exists when a particular stimulus is painful on the non-affected side, but not as strong as on the affected side [37]. The distinction between allodynia, which occur not only in response to mechanical stimuli but also to thermal stimuli [6], and hyperalgesia could not be sufficiently differentiated with the clinical tools and tests used in our study. In most cases, the participants reported no pain on the non-affected side, or rating 0/10 on the NRS. However, whether these observations would have altered the results cannot be determined definitively. According to the IASP there is no indication that allodynia (as frequently observed here, with participants reporting NRS 0/10 on the non-affected side) inherently signifies central sensitization [37]. Additionally, assessing or weighting a peripheral sensitization is more challenging in cases of extensive allodynia.

### Central sensitization

In addition to allodynia, extraterritorial sensitization and the wind-up ratio were considered relevant for weighting central sensitization. We found that the term ‘extraterritorial’ was defined with insufficient precision. Some raters indicated that testing has often followed along a peripheral nerve without comparing it to an area with a different sensory supply, while other raters focused more on the spread away from the original injury area. Similarly, determining the wind-up ratio posed challenges in individual cases. If patients exhibited high sensitization and experienced strong pain even with the first stimulus (e.g., NRS 7/10), the wind-up ratio could be biased because a maximum value of only ten could be given. Additionally, some tests had to be stopped prematurely due to pain. For instance, one subject aborted the test but only reported a value of NRS 3/10 at the end (starting at NRS 2/10). As a rater, one could subsequently interpret a high or low ratio, assuming that an abort actually means NRS 10/10. This example underscores the challenges of objectifying pain and the influence of subjective patient statements. Lastly, it is important to note that, to our knowledge, no study has yet investigated the validity of clinical sensory testing in patients with CRPS. However, Reimer et al. [38] proposed a bedside battery, but only 8% of their sample had CRPS.

### Cortical reorganization

The evaluation of cortical measures has prompted various questions and uncertainties. For instance, it is surprising to find that the threshold of two-point discrimination is lower in some participants on the affected side compared to the unaffected side. This finding contrasts with the common focus in existing literature on an elevated threshold [15, 22].

Furthermore, it is noticeable that the outcomes of the left-right recognition task do not consistently align with the described body perception. For instance, participants with IDs 6, 7, and 14 exhibited nearly flawless results in the Recognise™ App, yet reported entire limbs missing during their description of body perception to examiner A. Conversely, participants with IDs 15 and 17 showed minimal changes in body perception (e.g., slight thinning of fingers for ID 15, and equal and normal hands for ID 17), but their left-right recognition results, as per the NOI Group, fell below normal values [39]. It is conceivable that a learning effect occurs with frequent practice, without significant changes in cortical reorganization or subjective body perception. Our findings support the view that left-right recognition should be used only as an adjunct, in both assessment and treatment, with greater weight given to other measures of body perception disturbances, as suggested by other authors [40, 41].

Overall, the results of the BBPDS questionnaire revealed minimal variation in the scores obtained within our participant group. However, individuals with severe body perception disorders scored at least 25 out of 57 (refer to Table 2). Recent research by another group demonstrated good internal consistency and construct validity for the BBPDS. Nevertheless, they suggested removing the question concerning limb attention [42]. To our knowledge, no cutoff values or interpretations of the scoring from BBPDS have been investigated thus far.

In our study, relatively little attention was given to any loss of sensory function. This aspect was only locally tested in each case, primarily to facilitate the subsequent two-point discrimination assessment. Overall, findings on body perception are inconsistent, and mechanisms related to somatosensory functions and perceptions are still not fully understood [43]. Based on these considerations, we recommend prioritizing body perception assessments such as the BBPDS questionnaire. Newer tasks like Temporal Order Judgement (TOJ) or Apparent Body Movement Perception may be more appropriate in the future than laterality tasks, once accessible and standardized programs become available [41, 44].

### Vegetative responses

Furthermore, it is questionable whether the altered hair and nail growth should also be attributed to autonomic reactions. The autonomic nervous system regulates the formation of hairs (goose bumps) [45], but not their growth. Essentially, the physiological predisposition is hormone-dependent [46]. The mechanism of altered hair growth is not fully understood in CRPS, but neurogenic inflammation may play a part in it [47]. There is evidence that increased CGRP [4] or bone-building protein and increased blood flow [48] are associated with hyperhidrosis or -trichosis. Distinguishing between peripheral sensitization and vegetative responses is challenging since signs such as heating, redness, or swelling can be attributed to both mechanisms [49]. Despite this overlap, we recommend retaining the clinical signs assessed in this study as clinically feasible and relevant indicators.

### Statistics

In our study, we employed a pioneering methodology for analyzing compositional data as proposed by Chen [27], leveraging Bayesian statistics to quantify agreement among raters. To the best of our knowledge, this innovative approach has not yet been applied in this particular context, marking our endeavor as a potentially significant contribution to the field. While this method offers a robust and interpretable framework for assessing agreement, it assumes an ordinal relationship between categories – unlike our four distinct, non-hierarchical mechanisms. Despite this limitation, it remains the most suitable approach – to the best of our knowledge – currently available for analyzing compositional agreement in this setting.

Due to time constraints, we were only able to include 20 participants instead of the planned 30. While this reduced statistical power, we chose to report the findings given the methodological novelty and the relevance of our exploratory results.

### General strength and limitations

To our knowledge, our research is the only one to date that aimed at a mechanism-based classification of CRPS Type I signs with percentages for therapeutic purposes. Two other large studies [44, 50], similar to ours, attempted to divide CRPS patients into subgroups by clinical signs. One performed a dichotomous-classification based on objective signs of the Budapest criteria [50] while the other used QST, body perception assessments and psychosocial questionnaires [44]. Following their statistical analyses, both concluded that a clear classification into distinct subgroups is not possible and that the groups are highly heterogeneous. This supports our approach of not defining clearly distinguishable subgroups, but rather weighting pathophysiological mechanisms, as considerable overlap is common.

A general limitation of this study is the lack of agreement among all raters in two or three trials and their evaluation before the study commenced. Some difficulties could have been identified and addressed beforehand. Particular challenges were encountered in the interpretation of pain-provocating tests, as illustrated by the wind-up ratio example described above. Subjective patient responses may complicate clinical reasoning and affect agreement studies. However, the variability of the raters and the sample is considered a strength. Similarly, the participants exhibited varying complexities of clinical signs, with variable CRPS severity score and symptom durations.

While our findings showed good agreement among raters, it is important to clarify that our study did not assess inter-rater reliability in the traditional sense of independent clinical evaluations. Instead, agreement was based on standardized video and data collection. Thus, our results reflect consistency in classification and clinical reasoning, rather than full procedural reliability.

### Implications for practice

The complete assessment takes about 30 min. The identified dominant mechanisms could serve as an orientation for treatment planning. However, it should be noted that neither the proposed division into these mechanisms nor the effectiveness of this approach has yet been evaluated. If time is limited, we suggest prioritizing the CPS over the HPS, omitting the laterality recognition task, and simply asking patients to describe their body image with eyes closed (analogous to question 7 of the BBPDS). Clinical decision-making should not be based on a single assessment alone, but on the recognition of an overall clinical pattern, integration from multiple sources and flexibly adapting decisions to individual patients [51, 52].

#### Future research

For future studies with similar objectives to this one, it is crucial to first define terms more precisely such as “allodynia” and “extraterritorial”, which have posed challenges in this study. Similarly, clear requirements should be established for performing a wind-up ratio test. Additionally, there is a need for validation of clinical sensory testing in patients with CRPS. A follow-up study to this research could evaluate the clinically predominant complaint patterns identified in our mechanism-based classification and differentiate their respective clear positive assessments. It remains open whether this mechanism-oriented assessment is valid or requires reconsideration, particularly due to overlapping mechanisms. Of particular interest would be examining whether this assessment tested here and subsequent physiotherapeutic interventions would lead to improved outcomes in terms of pain and disability. For instance, treating patients with dominant cortical reorganization issues with a ‘cognitive multisensory rehabilitation’ and those with dominant central sensitizations mechanisms with guided graded exposure exercises, global endurance training and relaxing techniques. Although inter-rater agreement was generally good, percentage-based weighting may have added unnecessary complexity. A simplified ordinal scale (e.g. “low”, “moderate”, or “high”) could improve usability while preserving the intent of mechanism-based subgrouping in CRPS Type I.

As there are more conditions with several underlying mechanisms, the statistical method according to Bhatthacharyya coefficient for calculating intertester agreement can be applied to other areas of physiotherapy, such as non-specific low back or neck pain.

## Conclusions

Our exploratory study, aimed at evaluating the agreement of a physiotherapeutic assessment tool for CRPS Type I patients, yielded positive results, as indicated by the Bhatthacharyya coefficient. Despite of some difficulties, this work, with a detailed test protocol, presents a first proposal for assessments that could be conducted in a physiotherapeutic setting and how their positive findings could be interpreted. A clinical reasoning process is essential for developing individual and effective treatment strategies. However, it is important to note that the multifactorial pathophysiology and overlap of mechanisms in CRPS pose a major challenge in determining the primary driving mechanism and planning a subsequent treatment. Further research is necessary to establish consensus on a mechanism-based examination and its significance for clinical reasoning and physiotherapeutic treatment. Thus, we hope to contribute to a better understanding within physiotherapeutic institutions regarding physical examination and management in these rare and complex patients.

## Data Availability

No datasets were generated or analysed during the current study.

## Abbreviations

AROM: Active range of motion
BBPDS: The Bath CRPS body perception disturbance scale
BC: Bhattacharyya coefficient
CGRP: Calcitonin gene- related peptide
CM: Centimeter
CPS: Cold pain stimulation
CRPS: Complex regional pain syndrome
CSS: CRPS severity score
CST: Clinical sensory battery test
HPS: Heat pain stimulation
IASP: International association for the study of pain
ICC: Intraclass correlation coefficient
MM: Millimeter
MPS: Mechanical pain stimulation
NRS: Numeric rating scale
PPS: Pressure pain stimulation
QST: Quantitative sensory test
